# Radical Cure of Fistula in Ano and Hemorrhoids by Electricity

**Published:** 1889-10

**Authors:** W. S. Shotwell

**Affiliations:** Mich.


### Southern Medical Record.

A MONTHLY JOURNAL OF PRACTICAL MEDICINE.

☞ All communications must be addressed to R. C. WORD, Managing Editor.

"*Quicquid Præcipies Esto Brevis.*"

VOL. XIX ATLANTA, GA., OCTOBER, 1889. NO. 10

### ORIGINAL AND SELECTED ARTICLES.

### RADICAL CURE OF FISTULA IN ANO AND HEMORRHOIDS BY ELECTRICITY.

BY W. S. SHOTWELL, M. D., OF MICH.

I would call the attention of the profession to more rapid methods of curing fistula and hemorrhoids, coupled with safety and their radical extermination.

Having devoted years to this branch of the healing art, many times with tedious and unsatisfactory results, employing the much talked of Brinkerhoff, and other methods. I now challenge the world to compare results with the methods I now use in the cure of fistula in ano, be there one or a dozen openings. I employ an electrolytic battery of about 12 ampere power, with sufficient of the cautery element to subdue any hemorrhage that may perchance occur. My portable battery that I take to the patient's house is about 6 inches by 10 long and 10 inches high, with 2 cells, and built chiefly for quantity, charging it with tri oxide of chromium and sulphuric acid. The method of procedure is this: The battery is first charged, and the patient's bowels thoroughly emptied by means of an astringent injection. I then place the patient on his side, and with the Shotwell rectoscope or other suitable specula, the inner opening is located, or if it be an external incomplete fistula, the side opening of the rectoscope is so turned that the possible opening is in view. The patient is of course under the influence of an anæsthetic. I then straighten out the fistulous track nearest the anus, with a stiff steel probe of sufficient length, having an eye near its introductory end; and if the anus does not quite open into the bowel, perforate the intervening tissue till the eye of the probe is distinctly seen in the rectoscope, and leaving it there I next introduce a lance pointed probe, having also an eye near its end about three eighths of an inch farther from the anus into the solid structure and parallel with the fistulous track till its eye is also seen penetrating the bowel in the opening of the rectoscope. The eyès of both probes are then threaded with the opposite ends of a No. 24 platinum wire about 10 inches in length, and both probes are then withdrawn, leaving the wire *in situ*, forming a loop; both ends are now secured to an electrode, the electric current turned on and the loop drawn through the partition, in its passages destroying the membrane which lines the fistulous track. No dressing is necessary, as it is well known that no wound heals more kindly than those made by a battery. The bowels, however, must be kept locked up for at least a week, longer is better—when the patient gets up a well man, complete union taking place by first intention. The above method I have employed in many instances with complete success. For hæmorrhoids and prolapsus ani, I employ a similar treatment, no matter how large the protrusion or how long the patient has suffered; first bringing the growth all outside the anus, and in one treatment of a few moments the work is done and is always successful, followed by no hemorrhage or unpleasant symptoms or pain. And should your many readers desire further information, I shall be only too glad to give the same gra- tis to all who may apply.

